# Platelet-Rich Plasma Ameliorates Monosodium Iodoacetate-Induced Ankle Osteoarthritis in the Rat Model via Suppression of Inflammation and Oxidative Stress

**DOI:** 10.1155/2021/6692432

**Published:** 2021-01-18

**Authors:** G. H. Ragab, F. M. Halfaya, O. M. Ahmed, W. Abou El-Kheir, E. A. Mahdi, T. M. Ali, M. M. Almehmadi, U. Hagag

**Affiliations:** ^1^Anesthesiology and Radiology Department, Faculty of Veterinary Medicine, Beni-Suef University, Beni-Suef, Egypt; ^2^Physiology Division, Zoology Department, Faculty of Science, Beni-Suef University, P.O. Box 62521, Beni-Suef, Egypt; ^3^Department of Immunology, Military Medical Academy, Cairo, Egypt; ^4^Pathology Department, Faculty of Veterinary Medicine, Beni-Suef University, Beni-Suef, Egypt; ^5^Physiology Department, College of Medicine, Taif University, Taif, Saudi Arabia; ^6^Physiology Department, Faculty of Medicine, Beni-Suef University, Beni-Suef, Egypt; ^7^Department of Clinical Laboratory Sciences, College of Applied Medical Sciences, Taif University, P.O. Box 11099, Taif 21944, Saudi Arabia

## Abstract

Until now, there is no treatment that cause complete cure of the chronic inflammatory and degenerative disease, osteoarthritis (OA). Moreover, the underlying mechanisms of OA development and progress are not fully elucidated, and the present pharmacological treatment alternatives are restricted and associated with adverse side effects. Thus, the present study was conducted to evaluate the role of platelet-rich plasma (PRP) in the remedy of OA in the rat model in terms of inflammation, ankle histopathological alterations, and oxidative stress. OA was induced in male Wistar rats by injection of MIA (2 mg)/50 *µ*L isotonic saline in the right ankle joint for two successive days in each rat. After the 2^nd^ MIA injection, the osteoarthritic rats were allocated into two groups such as the MIA group (group 2) and MIA + PRP group (group 3). The MIA + PRP group was treated with PRP (50 *µ*L) by injection into the ankle joint of the right hind limb of each rat at days 14, 21, and 28 after the 2^nd^ injection of MIA. The same equivalent volume of saline, as a substitute of PRP, was injected into the ankle joint of each rat of the normal control group (group 1) and MIA group (group 2) at the same tested periods. Swelling of joint, bodyweight, total leucocytes count (TLC), and morphological as well as histological changes of ankle joints were evaluated. Serum lipid peroxides (LPO), glutathione (GSH), and glutathione S-transferase (GST) levels were examined as biomarkers of oxidative stress. Serum tumor necrosis factor-*α* (TNF-*α*), interleukin-17 (IL-17), and interleukin-4 (IL-4) were investigated by ELISA as biomarkers of inflammation. In addition, magnetic resonance imaging (MRI) was carried out to investigate the soft tissues in joints. The obtained results revealed that PRP reduced LPO and increased GSH and GST levels in osteoarthritic rats. Also, PRP significantly diminished serum TNF-*α* and IL-17 levels, while it increased IL-4 serum levels in rats with MIA-induced OA. Morphological observations, histological analysis, and MRI revealed a gradual diminishing in joint inflammation and destruction of cartilage in PRP-injected osteoarthritic rats. Based on these results, it can be suggested that PRP has antiarthritic potential in MIA-induced OA, which may be mediated via suppression of inflammation and oxidative stress.

## 1. Introduction

Osteoarthritis (OA), the main pervasive and destructive joint maladies, is a chronic inflammatory joint disease, which is characterized by alterations in synovial membrane, loss of joint cartilage, thickening of the joint capsule, and finally leading to pain, lameness due to stiffness of joints [1].

OA is a main reason of lameness and a popular trouble in all types of animal especially equine and pet animals. It can influence various joints. In performance and racing equines, it frequently influences the high mobility joints such as fetlock and carpal joints; although in equines utilized for less hard activities, it is more popular in the low motion joints, for example, the distal tarsal and pastern joints [2].

OA is initiated by several causes, and though elderly, it is the utmost common cause related to the OA progress; other etiological factors such as mechanical and hereditary factors also lead to OA progress. Moreover, OA is distinguished by the gradual damage of articular cartilage and osteophytes formation and is related to cartilage deterioration and subchondral bone alterations, which produce long-lasting pain and functional restrictions in the joint [3].

Although now accessible, clinical treatments for OA that incorporates usual analgesics and calming nonsteroidal anti-inflammatory drugs (NSAIDs) are unavailing in decelerating disease development, and they slightly improve signs by diminishing pain and increasing joint motion. Furthermore, their long-lasting usage has been restricted by their harmful aspect effects, and surgical interferences are ultimately needed [4].

Platelets-rich plasma (PRP) acts like a biologic incentive to affect cartilage restoration. Despite the verity that the mixture of growth factors essential to the PRP regenerative properties is ambiguous, the transforming growth factor-*β*1 (TCF-*β*1) has been proposed to promote stem cells, proliferation of chondrocyte, and restrict catabolic action [5].

PRP has achieved publicity as a clinical treatment in soft and hard tissues in all surgical fields, most prominently in acute surgical conditions and in the lasting wound management. Surgeons are utilizing PRP to take benefit of fibrin clot that help in hemostasis accompanied by growth factors supplying in this form to enhance wound healing [6].

The accomplishment of this curative sits is not only restricted to the characteristic of PRP but also to its reliable treatment. Improper use of PRP can promote an ineffectual biological reply and inappropriate clinical outcomes. Thus, intraarticular injection that extends to the cartilage and the synovial membrane successfully improves the joint environment, slows joint pain progression, and adjusts the clinical symptoms [7].

Therefore, the purpose of the existing work was to assess the efficacy of intraarticular ankle injection of PRP in ameliorating inflammation, joint damage, and oxidative stress induced by monosodium iodoacetate- (MIA-) induced ankle OA in the rat model.

## 2. Materials and Methods

### 2.1. Animals

Thirty male Wistar rats were used in the current investigation. Their weights ranged from 100 g to 120 g, being 7–9 weeks of age. The animals were obtained from the Laboratory Animal Unit of Helwan Farm, Holding Company for Biological Products and Vaccines (VACSERA), Egypt. Animals were retained under observance for about 10 days prior to the beginning of the research to eradicate any infections. The animals were kept in cages made from polypropylene with ventilated covers of stainless steel in the Animal House of Department of Zoology, Faculty of Science, Beni-Suef University, Egypt, at standard temperature (20–25°C) and ordinary daily lighting cycle (10–12 h/day) and were supplemented balanced standard diet and water ad libitum.

### 2.2. Induction of Osteoarthritis

Under anesthesia using ketamine (70 mg/kg) and xylazine (7 mg/kg), OA was induced by injecting 50 *μ*L physiological saline containing 2 mg MIA (2 mg/50 *μ*L) (Sigma-Aldrich, St. Louis, MO) with a 21-gauge needle into the ankle joint of the right hind leg on 2 succeeding days, as formerly illustrated [8].

### 2.3. PRP Preparation

PRP was prepared using the double spin method in accordance with the manner of Pacheco et al. [9] and Asjid et al. [10] with some modifications. Blood was collected by puncture of the heart of 5 healthy rats and kept in tubes with anticoagulant (3.8% sodium citrate). The technique was performed under sterile condition, in a Biobase vertical laminar flow cabinet (Biobase model: BBS V1300; NO-51, South Gongye Road, Jinan, Shandong Province, China). Lysing platelets were averted to preclude their ability loss to excrete growth factors. Samples of blood were centrifuged at 1000 round per minute (rpm) for ten minutes to separate RBCs, WBCs, and platelet cells. The upper part of the supernatant, up to the fog zone edge, which corresponds to plasma and platelets, was collected into new tubes. PRP was obtained by centrifugation of these tubes at 2000 rpm for 10 minutes and disposal of the supernatant, merely the lower 20% of the plasma was reaped (PRP or plasma rich in platelets). Around, the upper 80% of the plasma was taken away and kept into another tube considered as PPP (plasma poor in platelets). The residual material including the platelet pellet was resuspended, producing the PRP that was deemed appropriate for the study's aim. PRP prepared in this experiment was utilized within 6 hours. PRP was activated by adding 50 *μ*L 10% calcium chloride (LABiTec GmH, Germany) (0.025 mol/L) to 3 ml blood. PRP was administrated by intraarticular injection immediately after activation.

### 2.4. Animal Grouping

After the accommodation period, the Wistar rats were randomly allocated into three groups (10 rats/each group).

#### 2.4.1. Normal Control Group

It is composed of normal rats that were injected with 50 *µ*L isotonic sterile saline in the ankle joint of the right hind limb of each rat at 14, 21, and 28 days.

#### 2.4.2. MIA Group

Rats in this osteoarthritic group were injected with MIA in the ankle joint of the right hind limb in two consecutive days. The rats within this group were also injected with 50 *µ*L isotonic sterile saline in the ankle joint of the right hind limb at 14, 21, and 28 days after MIA injection.

#### 2.4.3. MIA-PRP Group

This osteoarthritic group were injected with MIA in the ankle joint of the right hind limb in two consecutive days and also injected with PRP (50 *µ*L) into the ankle joint of the right hind limb at 14, 21, and 28 days after injection of MIA.

The bodyweight was measured once a week. At the end of experimental periods, under diethyl ether anesthesia, we collected blood samples from jugular vein. A portion of blood from every rat was collected in tubes having ethylenediamine tetra acetic acid (EDTA) solution (50 ml of 15% EDTA/2.5 ml blood) for leukocytes count. Another portion of blood was collected in tubes having no anticoagulant and allowed to coagulate and then centrifuged at 3000 rpm for 15 min. The clear nonhaemolysed supernatant sera were quickly aspirated and preserved at −20°C until utilized.

### 2.5. Ankle Measurement

The alterations in the transverse and anteroposterior diameters of the osteoarthritic and normal ankles were observed. Ankle diameters were measured using a micrometer [11]. The measurements were recorded every week (on the day zero till the end of experiment) after MIA injection. Also, the right legs were photographed by a camera.

### 2.6. Magnetic Resonance Imaging (MRI)

The right hind legs of normal, MIA, and MIA-PRP Wistar rats were subjected to random scan by MRI before and after treatment. Rats were chosen from every group and scanned after anesthesia by ketamine and xylazine (70 mg/kg ketamine and 7 mg/kg xylazine). The rats were examined on a 1.5 Tesla whole body MR scanner (Philip Medical System, Intera) with an extremity coil. The rats were located sited in prone situation with the hind legs expanded caudolaterally through using tape to fix the rat, so that the right ankle joint was placed in the middle of the scanning coil. MR images were obtained with a sequence of T1 weighted in coronal slice orientation by the succeeding series parameters (TR = 3000 ms, TE = 15 ms, and slice thickness = 2 mm).

### 2.7. Detection of Total Leukocytes Count

TLC was assessed by using Turk's solution that composed of a stain (gentian violet) and 1% acetic acid [12].

### 2.8. Detection of Serum Cytokines

TNF-*α*, IL-17, and IL-4 levels were assayed by utilizing special ELISA (enzyme-linked immunosorbent assay) kits obtained from R and A systems, USA.

### 2.9. Detection of Serum Oxidative Stress and Antioxidant Defense Markers

Serum lipid peroxides (LPO) and glutathione (GSH) levels were detected based on the procedures of Preuss et al. [13] and Beutler et al. [14], respectively, with some minor alterations. The activity of serum glutathione S-transferase (GST) was determined in accordance with Mannervik et al. [15].

### 2.10. Histopathological Examination

After sacrifice (42 days after MIA injection), the right ankles were removed and placed in 10% buffered formalin for 48 hours. Decalcification was performed with 10% formic acid which was replaced twice weekly for two weeks. The end point of decalcification was assessed physically with a surgical blade. After complete decalcification, the samples were washed with phosphate buffer solution (PBS), dehydrated in a graded ethanol series, and embedded in paraffin wax. Sagittal sections measuring 5 *µ*m in thickness were prepared and stained with hematoxylin and eosin (H&E) [16]. Histopathological examination of synovial inflammation, cartilage, and bone damages were performed by a pathologist blindly.

### 2.11. Statistical Analysis

Statistical analysis was achieved by using SPSS v.25. Results were expressed as mean ± standard error (SE), and all statistical comparisons were performed by Duncan's test post hoc. Values of *p* < 0.05 were deemed significant; however, those of *p* > 0.05 were deemed nonsignificant.

## 3. Results

### 3.1. Morphological Feature

**Figure 1:**
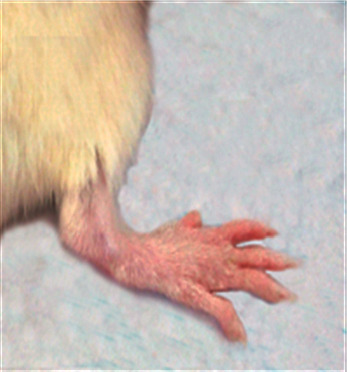
Ankle joint of normal control rat.

**Figure 2:**
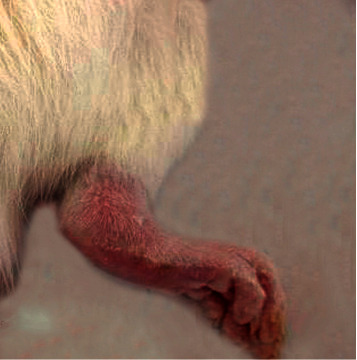
Ankle joint of osteoarthritic rat on 1^st^ week.

**Figure 3:**
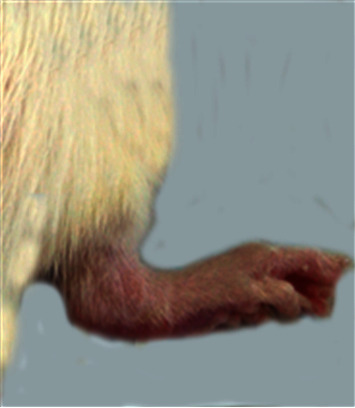
Ankle joint of osteoarthritic rat on 6^th^ week.

**Figure 4:**
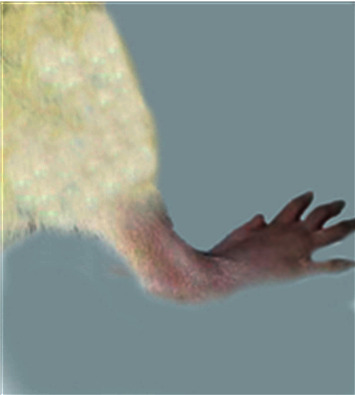
Ankle joint of osteoarthritic rat treated on PRP 6^th^ week.

The morphological alterations in the right ankles of the normal control, osteoarthritic group (MIA group), and osteoarthritic-treated group (MIA + PRP group) are shown in Figures 1–4. The right legs showed noticeable swelling and redness at the 1^st^ week (Figure 2) and 6^th^ week (Figure 3) after injection of MIA when compared with those of normal control groups (Figure 1). These worsened signs were more distinct at the 1^st^ week (acute inflammation). The remedy of osteoarthritic rats with PRP resulted in a significant improvement of these morphological symptoms as shown in Figure 4 (at the 6^st^ week).

### 3.2. Effect on Bodyweight

**Figure 5:**
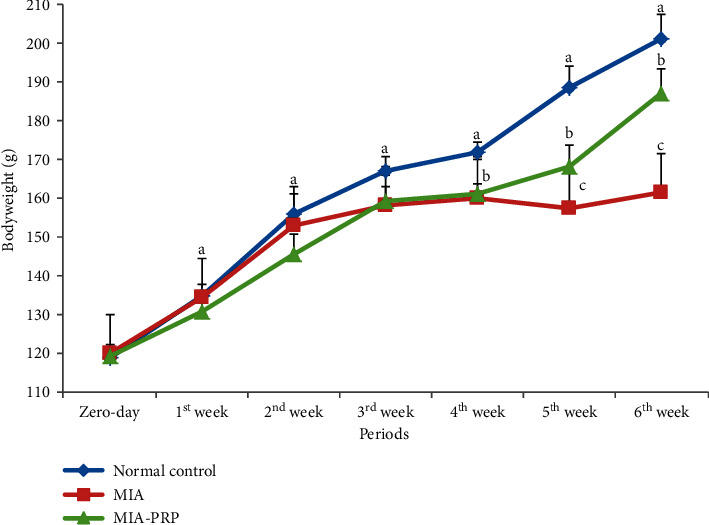
Bodyweight changes in normal control, MIA, and MIA-PRP groups. At each period, the means, which have different symbols (letters), are significantly different at *p* < 0.05.

The changes of bodyweight in the normal control, MIA-administered group, and MID + PRP-administered group through six weeks after MIA administration are shown in Figure 5. The MIA-administered group exhibited a significant decrease (*p* < 0.05) in the bodyweight at periods 4, 5, and 6 weeks; the recorded percentage decreases were −6.8%, −16.5%, and −19.8%, respectively, as compared to the normal control group.

The remedy of the osteoarthritic rats with PRP produced a significant increase (*p* < 0.05) in bodyweight at the 5^th^ and 6^th^ weeks; the recording percentage changes were 6.9% and 15.9% in comparison with the MIA group.

### 3.3. Alterations in Ankle Swelling Indices

**Figure 6:**
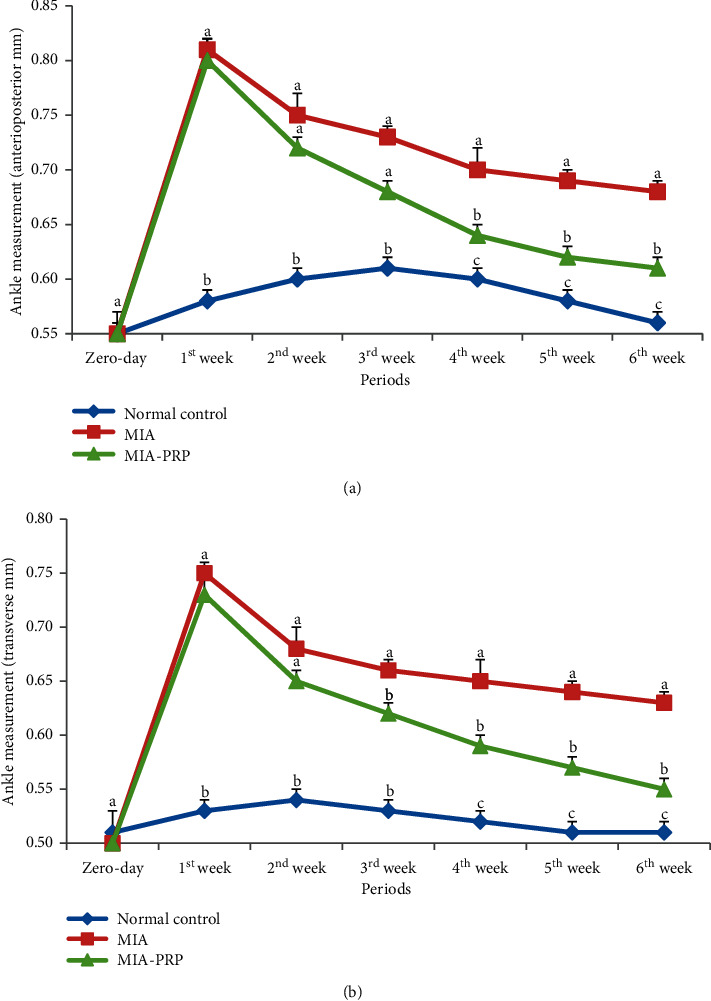
(a) Ankle measurements (anteroposterior) in normal control, MIA, and MIA-PRP groups. At each period, the means, which have different symbols (letters), are significantly different at *p* < 0.05. (b) Ankle measurements (transverse) in normal control, MIA, and MIA-PRP groups. At each period, the means, which have different symbols (letters), are significantly different at *p* < 0.05.

As compared with normal control animals, MIA rats exhibited a significant increase in the right ankle anteroposterior and transverse diameters at all check periods except at zero time (Figures 6(a) and 6(b)). On the other hand, the MIA + PRP group exhibited a marked decrease in the right ankle anteroposterior and transverse diameters at all check timepoints after MIA injection. The effect PRP on anteroposterior diameter was significant at the 4^th^, 5^th^, and 6^th^ weeks after MIA injection, while the effect on transverse diameter at the 3^rd^, 4^th^, 5^th^, and 6^th^ weeks in comparison with MIA control. The ameliorating effects were more pronounced at the period extended to 6 weeks. Hence, PRP treatment yielded obvious influences on the swelling rate of ankle.

### 3.4. MRI Evaluation of OA

**Figure 7:**
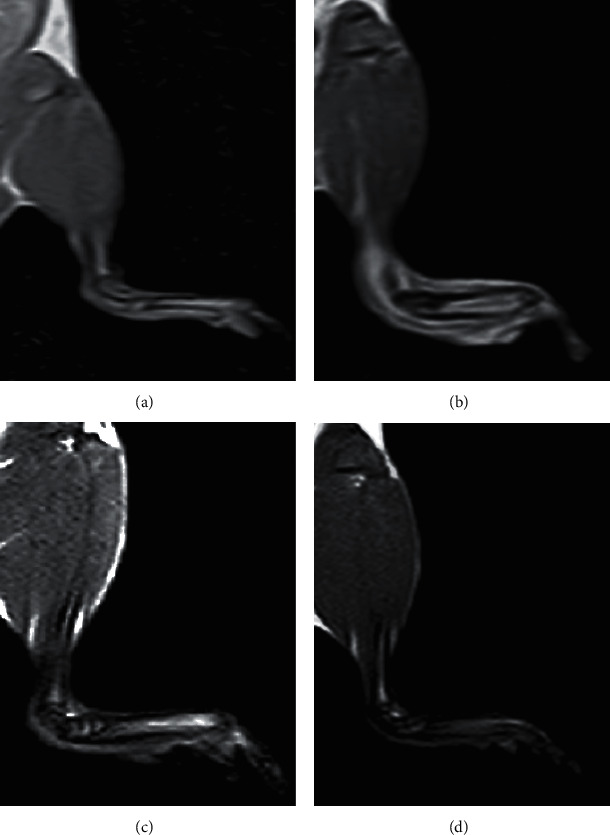
T1-weighted MR images of the right ankle joints of normal control, MIA, and MIA + PRP groups showing normal joint and foot anatomy (Figure 7(a)), enlarged diameter of the joint with extensive soft-tissue edema in acute osteoarthritic rats (Figure 7(b)), and reduced soft tissue edema in chronic osteoarthritic rats and still enlarged joint diameter as compared to the normal control (Figure 7(c)). In contrast, PRP treatment revealed a diminished diameter of the joint resembling that of normal control (Figure 7(d)). (a) Normal control. (b) Acute OA (MIA group). (c) Chronic OA (MIA group). (d) MIA-PRP group.

MRI of the normal ankle joint demonstrating normal anatomy of the joint and foot is shown in Figure 7(a). On the other hand, MRI of an osteoarthritic ankle joint after MIA injection reflects the increased diameter of the joint and displays extensive soft tissue edema in acute osteoarthritis (Figure 7(b)) and soft tissue edema decreased in chronic osteoarthritis (Figure 7(c)). In contrast, the treatment with PRP exhibited mostly low signals and diminished diameter of the joint (Figure 7(d)), showing an effective suppression of inflammation and curative outcome.

### 3.5. Effect on TLC

**Figure 8:**
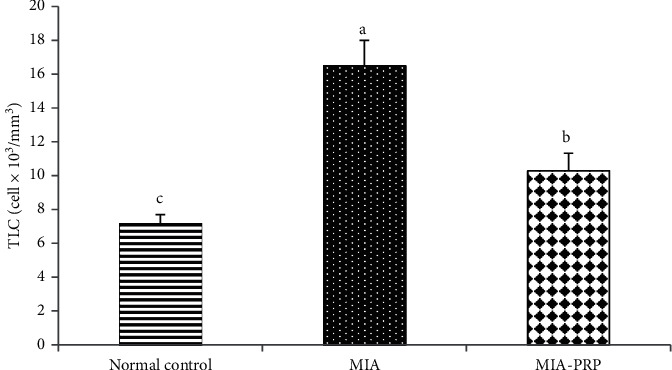
TLC in normal control, MIA, and MIA-PRP groups. The means, which have different symbols (letters), are significantly different at *p* < 0.05.

TLC was significantly raised (*p* < 0.05) in the MIA-induced osteoarthritic group when compared with the normal control group. Osteoarthritic rat's treatment with PRP resulted in a marked improvement (*p* < 0.05) in TLC (Figure 8).

### 3.6. Effect on Serum TNF-*α* (Th1 Cytokine), IL-17 (Th17 Cytokine), and IL-4 (Th2 Cytokine) Levels

**Figure 9:**
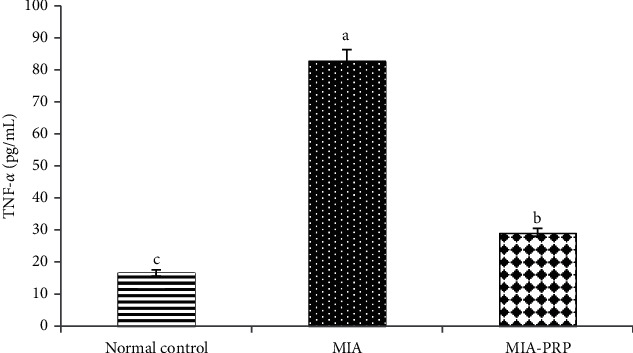
Serum TNF-*α* level in normal control, MIA, and MIA-PRP groups. The means, which have different symbols (letters), are significantly different at *p* < 0.05.

**Figure 10:**
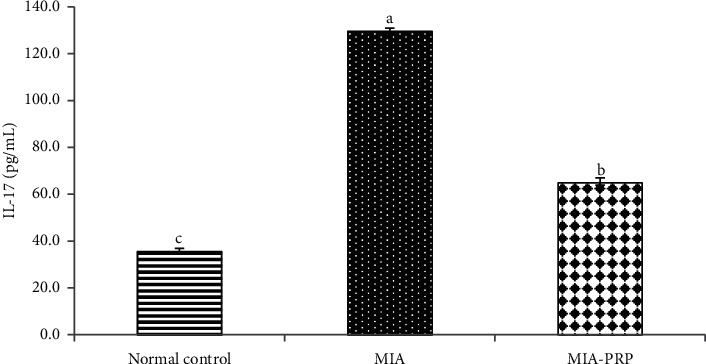
Serum IL-17 level in normal control, MIA, and MIA-PRP groups. The means, which have different symbols (letters), are significantly different at *p* < 0.05.

**Figure 11:**
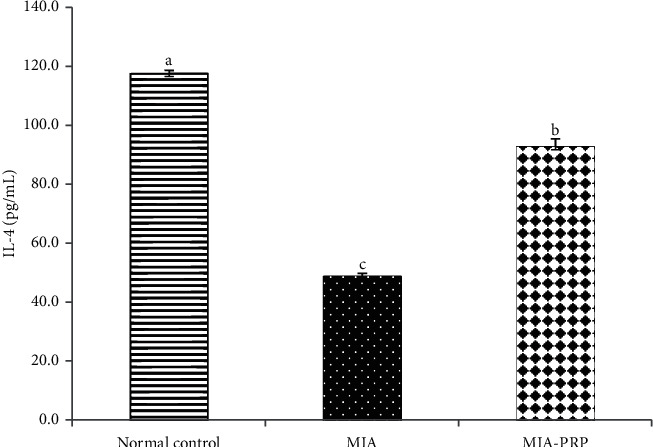
Serum IL-4 level in normal control, MIA, and MIA-PRP groups. The means, which have different symbols (letters), are significantly different at *p* < 0.05.

The serum TNF-*α* and IL-17 levels were significantly (*p* < 0.05) increased in MIA-induced osteoarthritic rats when compared to normal control rats. The remedy of MIA-induced osteoarthritic rats with PRP resulted in a significant (*p* < 0.05) reduction of the raised TNF-*α* and IL-17 levels (Figures 9 and 10). In contrast to TNF-*α* and IL-17, the IL-4 level in serum was extensively lessened (*p* < 0.05) in MIA-induced osteoarthritic rats. The remedy of osteoarthritic rats with PRP markedly boosted (*p* < 0.05) the lessened IL-4 level (Figure 11).

### 3.7. Effect on Antioxidant Defense and Oxidative Stress

Administration of MIA significantly elevated serum oxidative stress as evidenced by the significant increase (*p* < 0.05) in the serum LPO level and obvious lessening (*p* < 0.05) in the serum GSH level and GST activity when compared to normal control rats. Treatment with PRP hindered oxidative stress induced by MIA as recognized by marked decrease (*p* < 0.05) in the serum LPO level and raises (*p* < 0.05) of the diminished serum GSH level and GST activity when compared to the MIA group; hence, PRP diminished oxidative stress and enhanced antioxidant defense mechanism (Table 1).

Data are expressed as mean ± standard error. Number of noticed samples in every group is 6. Means, which have the similar superscript symbol (s), are not significantly different. Percentage changes were estimated by the MIA group with the normal control group and the PRP group with the MIA group.

### 3.8. Histopathological Changes

**Figure 12:**
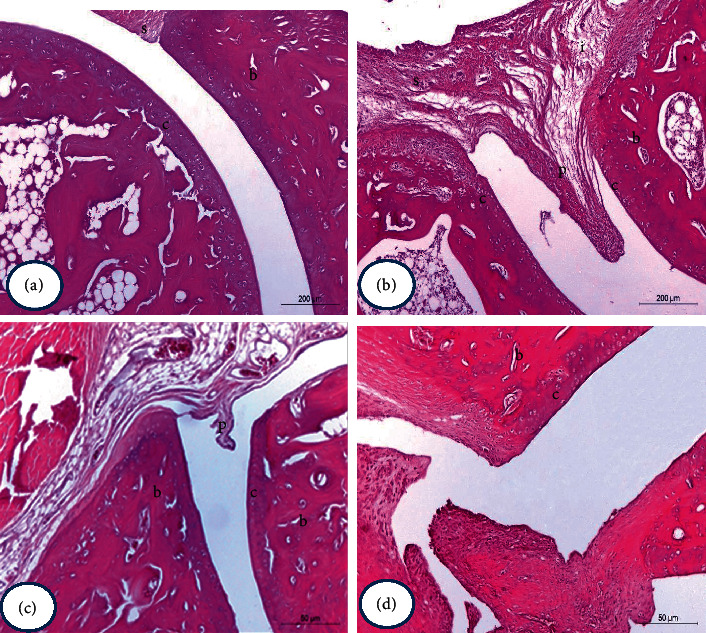
Photomicrographs of H&E-stained sections of the hind right leg ankle of normal control (a), MIA rats (b), and MIA + PRP rats (c and d) (H&E X100). The normal histological image (a) showed the normal histological structure of the synovial membrane (s), articular cartilage (c), and bone (b). Photomicrographs of ankle joint of MIA rats illustrated hyperplasia of synovial membrane (s), infiltration of inflammatory cells (i), marked pannus formation (p), damage of cartilage (c), and bone erosion (b). The photomicrographs of hind ankle joints of PRP-treated rats (c and d) revealed mild to moderate arthritis pathology, respectively.

Hematoxylin and eosin-stained sections of ankle joint tissues from normal control rats revealed no inflammation and normal histological structure of the joint (bone, cartilage, and fibrous joint capsule) (Figure 12(a)). However, stained sections of osteoarthritic control rats (MIA) revealed marked histopathological changes in the form of synovial hyperplasia with infiltration of a large number of inflammatory cells (lymphocytes, macrophages, and sometimes plasma cells), extensive pannus formation, and severe cartilage and bone destruction (Figure 12(b)). On the other hand, osteoarthritic rats treated with PRP showed mild to moderate degree of osteoarthritis (Figures 12(c) and 12(d)). Microscopically, MIA rats showed synovitis characterized by proliferating synovial lining cells, in 2-3 layers, as well as proliferation of the underlying blood vessels, which was associated with perivascular edema and diffused cellular infiltration composed of mononuclear cells. In many tissue specimens, the inflammatory cellular exudate extended to involve the whole periarticular soft tissues such as connective tissue and muscles. There was synovial sloughing in some areas of synovial membrane and mild proliferative lesions of fibroblast-like cells. Pannus formation was in the form of single or multiple proliferating granulation tissue containing hyperplastic synoviocytes and inflammatory cells at the articular cartilage margin and at the cartilage-bone level. The articular cartilages of some arthritic rats had uneven articular surface and demonstrated superficial fibrillation accompanied by cell death or proliferation and in some cases extended to the midzone portion of the articular cartilage. Moreover, the articular bone destruction was visualized by osteoclast activity and fibroplasia. However, osteoarthritic rats treated with PRP showed the previously mentioned histopathological lesions of arthritis but with mild to moderate degree.

## 4. Discussion

OA is a lasting progressive joint disease. Its origin is multifactorial and characterized by gradual articular cartilage damage, subchondral bone sclerosis, and synovitis [17]. Existing therapy alternatives involve analgesics, intraarticular hyaluronic acid, corticosteroid, NSAIDs, and PRP injection as well as physical treatment and surgical interferences [18].

Therefore, in the current investigation, the influence of intraarticular PRP administration on MIA-induced osteoarthritic rats was evaluated, and the roles of oxidative stress, antioxidant defense mechanism, and the inflammatory status were scrutinized.

MIA-induced osteoarthritis is a usually used experimental model for preclinical investigations. Because the duration of testing is short, its application is simple, and it is similar to animal and human OA, and this model is used commonly to assess curative agents [19]. In our study, the bodyweight loss is used as the clinical outcome associated with OA. The osteoarthritic rats showed a significant decrease in the bodyweight at the 4^th^, 5^th^, and 6^th^ weeks when compared to the normal control rats. These results are in accordance with the previous study, which reported that progressive lessening of bodyweight has been achieved between arthritic animals throughout the progress of arthritis [20]. Also, it was reported that the injection of MIA caused a marked reduction in bodyweight when compared with normal animals [21]. No obvious variations were noticed in bodyweight between the MIA-induced OA and normal control at the first 3 weeks, while the PRP-treated group exhibited a significantly (*p* < 0.05) higher bodyweight than the MIA group at the 5^th^ and 6^th^ weeks. The bodyweight rate elevated in this period, proposing that the rats were under fewer stress and/or in fewer pain.

In the present study, significant increases in both the right ankle anteroposterior and transverse diameters in the MIA group were noticed at all periods after MIA administration relative to the normal control group. These results are in accordance with previous publications that revealed that MIA injection increased the ankle anteroposterior and transverse diameters [22]. In the current study, PRP produced a significant decrease in the elevated values of the right ankle anteroposterior and transverse diameters when compared to MIA animals after the 4^th^ and 3^rd^ weeks, respectively. In parallel with this study, Aniss et al. (2020) stated that the treatment of rats with PRP for six weeks in CFA-induced arthritis results in the decline of paw swelling [23].

Over the previous years, the diagnostic use of MRI in the osteoarthritis study has advanced from a technique to one of the applications for imagining of soft tissue and changes of the bone in arthritic joints [24]. The synovial membrane of arthritic rats with early OA is characterized with hyperplasia and increased vascularization. MRI also depicts hyperemia of the synovial membrane prior to damaging lesions of the cartilage and bone. However, the usual usage of MRI is restricted due to it is expensive and time consuming [25]. In this work, magnets with low field strength 1.5 Tesla lead to poor anatomic resolution. Extra shortage involved an incapability to illustrate the underlying pathology relating to alterations in hydrogen content in osteoarthritic joints. The final aim of this investigation was to assess the data of MRI in the perspective of a collection of physiologic (bodyweight and ankle measurement), biochemical (oxidative stress and cytokines), cellular (TLC), and parameters of histology. This assessment was not aimed at defining if MRI could replace for any one indicator of disease development but to define if alterations in MRI images could be related with any other systemic actions. In our study, boosts intensity of MRI signal in the right hind paw strongly reflected rises in ankle measurement and leucocytic counts. These last inflammatory replies peaked among days 3 and 14 after administration of MIA. After treatment of osteoarthritic rats with PRP, the intensity of the signals of MRI subside at 4–6^th^ week, similar to the reduction detected in ankle measurements and morphological changes, demonstrating that the inflammatory response was in diminution in osteoarthritic rats treated with PRP. The TLC data showed a profound leukocytosis in the MIA-induced osteoarthritic animals. This leukocytosis is attributed to inflammation induced by MIA [26]. In the existing study, it was found that in the PRP-treated osteoarthritic group, the elevated TLC declined markedly near to their normal levels. PRP has an anti-inflammatory effect which is mostly related to reduction in TLC [27]. New experiments have suggested a role of oxidative stress in the pathogenesis of OA.

**Figure 13:**
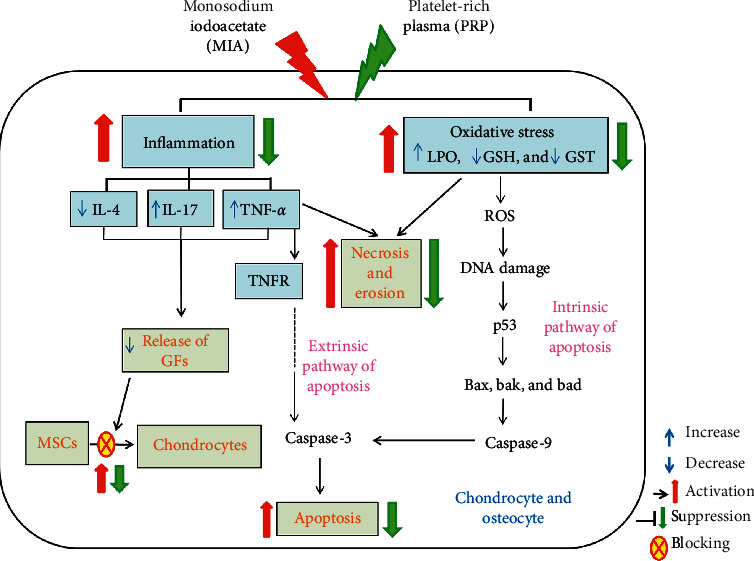
The roles of inflammation and oxidative stress in MIA-induced osteoarthritis and effects of treatment with PRP. GFs, growth factors; MSCs, mesenchymal stem cells; TNFR, tumor necrosis factor receptor.

Oxidative stress is always created, influencing cells and the extracellular matrix. Excessive ROS levels, in combination with the antioxidant reduction, take part in the development of disease (Figure 12) [28, 29]. In the current investigation, the induction of OA using MIA was produced via various mechanisms. One of these mechanisms was the beginning of oxidative stress as illustrated by marked increase in the serum LPO level in association with marked decrease in the serum GSH level and GST activity. This is in line with the observation of previous report, which found that MIA or its metabolites yield free radicals which attack lipid components, resulting in formation of LPO [30]. Amplified free radical production from inflammatory site leads to reinforced osteoarthritis and the decreased level of cellular antioxidant [31]. In the current study, the GSH level and GST activity in the MIA-induced osteoarthritic rats was significantly decreased as compared to the normal control rats. Similar effects on GSH and GST levels were revealed in plasma of MIA-induced osteoarthritic rats [32]. In the current investigation, intraarticular injection of PRP to MIA-induced osteoarthritic rats markedly diminished the serum LPO level, while it noticeably elevated the GSH and GST levels. These attained data confirmed the antioxidant characteristic of PRP. This effect has been studied in previous publications [33, 34] that stated that PRP might forbid oxidative stress via the incitation of the transcription nuclear erythroid 2-related factor (Nrf-2) antioxidant response element signaling. Furthermore, several growth factors released from PRP can stimulate T cell which can reduce ROS production and raise the resistance level to oxidation [35]. The increase in the oxidative stress stimulates DNA damage and expression of proapoptotic protein (p53); thus, it activates the intrinsic pathway of apoptosis [36] in addition to necrosis leading to cartilage erosion and bone damage (Figure 13).

Other suggested mechanism for MIA-induced osteoarthritis is the motivation of inflammatory cytokines. Inflammation and inflammatory response are considered as crucial factors that begin and hasten the OA development (Figure 13). It is extensively believed that inflammatory cytokines are essential mediators in the troubled metabolism and boosted tissue catabolism in OA joint [37]. The long-lasting inflammatory process is mediated via a complicated cytokine network [38]. In the OA pathogenesis, there is an important reason that inflammatory mechanisms play a vital role in OA [39]. In the current study, the concentrations of TNF-*α* and IL-17 (proinflammatory cytokines) in addition to IL-4 (anti-inflammatory cytokine) were detected in the serum of all rat groups to check the inflammatory status. Current data demonstrated that the MIA group exhibited an obvious increment in levels of TNF-*α* and IL-17 in rat serum and a significant decrease in the serum IL-4 level when compared to the normal control rats evidencing the inflammation induction in the joints of rats (Figure 13). Like to the existing results, former data proved that MIA significantly increased levels of IL-17 and TNF-*α* in serum of rats [40], whereas the serum IL-4 level was notably diminished after MIA injection as compared with normal control rats [41]. These increments in the concentrations of proinflammatory cytokines may mirror their critical role in the arthritis progress pathophysiology in animal models [42]. The MIA injection to the rats provokes the increase in the inflammatory cytokines, while it suppresses the anti-inflammatory cytokines, thereby developing the inflammation process. In addition to the necrotic effects of TNF-*α*, it activates the tumor necrosis factor receptor or death receptors, thereby activating the extrinsic pathway of apoptosis (Figure 13) [43, 44]. Furthermore, the inflammatory environment and the increased levels of TNF-*α* and IL-17 could result in a decrease in the formation and release of growth factors (GFs) such as transforming growth factor (TGF) leading to reduced chondrogenesis and formation of chondrocytes from mesenchymal stem cells (Figure 13) [45]. The osteoarthritic rats treated with PRP showed a significant decline in serum TNF-*α* and IL-17 levels when compared to the elevated level of the osteoarthritic control animals, while they exhibited a significant elevation of the lowered serum IL-4 level. Thus, PRP may counteract cartilage erosion by inhibiting the TNF-*α* (proinflammatory cytokine) and increasing the anti-inflammatory cytokine IL-4 level (Figure 13) [46, 47].

The pannus formation, degeneration of cartilage, synovial hyperplasia, and inflammation exhibited that the MIA-induced osteoarthritis model is closely similar to human OA [22]. Therefore, in the current work, a rat model of MIA-induced osteoarthritis was established and utilized by ankle intraarticular injection of MIA. MRI and histological examinations performed in this study exhibited that the OA rats exposed obvious deterioration of joint structure and reduced ankle joint space. Furthermore, according to analysis of histopathology results of the ankle joint, synovial hyperplasia, cartilage destruction, erosion of bone, and inflammatory cells were observed in the MIA rats. These phenomena were also illustrated in former studies [48, 49]. In the current study, the treatment with PRP obviously declined swelling of paw and osteoarthritis induced by MIA as compared with MIA control rats. This investigation further verified its curative effect by histopathological evaluation. It was evidenced effective in diminishing hyperplasia of synovial membrane, cartilage destruction, and bone erosion degree. These results are in agreement with the previous study [23].

## 5. Conclusion

Intraarticular injection of PRP offers trust for osteoarthritis improvement. Intraarticular PRP treatment diminishes manifestations of OA due to its anti-inflammatory effects and antioxidant effects. Though, additional studies are necessitated to evaluate PRP effectiveness in human beings.

## Figures and Tables

**Table 1:** Serum LPO, GSH level and GST activity in normal control, MIA, and MIA-PRP groups.

Groups	LPO (nmol/100 mL/hr)	%	GSH (×10^2^) (nmol/100 mL)	%	GST (nmol/L)	%
Normal	0.12 ± 0.02^c^	ـ	78 ± 8^a^	ــ	303 ± 40^a^	ـ
MIA	0.79 ± 0.05^a^	543	27 ± 6^b^	−100	130 ± 16^b^	−57
MIA + PRP	0.33 ± 0.06^b^	−64	93 ± 7^a^	250	298 ± 25^a^	130

## Data Availability

The data used to support the findings of this study are available from the corresponding author upon request.

## Additional Points

A main limitation is that the PRP utilized in this investigation was not characterized by its composition and physical characteristics. Besides variations between donors, consequently, the composition of the PRP isolated will differ from one individual to another in composition.
